# Metabolomic Signatures of Physical Function and Functional Trajectories in Older Adults: Insights from the ENRGISE Clinical Trial

**DOI:** 10.3390/metabo16010009

**Published:** 2025-12-22

**Authors:** David H. Lynch, Liubov Arbeeva, Susan C. J. Sumner, Blake R. Rushing, John A. Batsis, Amanda E. Nelson, Roger A. Fielding

**Affiliations:** 1 Division of Geriatric Medicine, Center for Aging and Health, University of North Carolina at Chapel Hill, Chapel Hill, NC 27599, USA; 2 Cecil G. Sheps Center for Health Services Research, University of North Carolina, Chapel Hill, NC 27599, USA; 3 Thurston Arthritis Research Center, School of Medicine, University of North Carolina at Chapel Hill, Chapel Hill, NC 27599, USA; 4 Nutrition Research Institute, Department of Nutrition, University of North Carolina, Kannapolis, NC 28081, USA; 5 Department of Nutrition, Gillings School of Global Public Health, University of North Carolina at Chapel Hill, Chapel Hill, NC 27599, USA; 6 Metabolism and Basic Biology of Aging Directive, Jean Mayer USDA Human Nutrition Research Center on Aging, Tufts University, Boston, MA 02111, USA

**Keywords:** aging, physical function, geroscience, metabolomoics

## Abstract

**Background:** Chronic inflammation contributes to functional decline in older adults, yet interventions targeting inflammatory pathways have shown inconsistent results. Metabolomics offers a promising approach to identify biological heterogeneity and uncover molecular signatures underlying differential functional trajectories. **Objective**: Our objective was to examine whether untargeted serum metabolomics can identify metabolic signatures associated with baseline physical function, functional trajectories, and treatment response in older adults with chronic inflammation participating in the ENRGISE trial. **Methods:** We performed untargeted metabolomic profiling on serum samples (n = 731) collected at baseline, 6, and 12 months from participants (mean age ≥ 70) enrolled in the ENRGISE pilot randomized trial. Participants were randomized to losartan, omega-3 supplementation, both, or placebo. Functional measures included grip strength and 400 m gait speed. Group-based trajectory modeling classified participants into functional trajectories over 12 months. Partial least squares-discriminant analysis (PLS-DA) and pathway enrichment (mummichog algorithm) were used to identify differentially abundant metabolites and perturbed pathways. **Results:** Baseline metabolomic profiles differed by physical function status. Participants with low grip strength showed enrichment in vitamin A metabolism pathways, while slower gait speed was associated with higher levels of prostaglandin and eicosanoid metabolites. Baseline metabolic profiles distinguished individuals who later declined versus improved in functional performance. Omega-3 supplementation, but not losartan, induced distinct changes in lipid-related pathways, including fatty acid activation, omega-3 metabolism, and prostaglandin biosynthesis, indicating that individuals responded to these interventions metabolically despite null clinical outcomes. **Conclusions:** Serum metabolomic signatures were associated with baseline physical function, predicted functional trajectories, and revealed pharmacologic activity of omega-3 supplementation. These findings support the use of metabolomics to uncover biological heterogeneity and inform precision geroscience strategies in aging populations.

## 1. Introduction

Maintaining physical function in older adults is critical for preserving independence and quality of life, as mobility limitations are strongly associated with higher risks of hospitalization, nursing home placement, and mortality [1,2,3]. Aging is often accompanied by chronic low-grade inflammation (termed “inflammaging”), characterized by elevated circulating cytokines such as interleukin-6 (IL-6) and C-reactive protein [4,5]. This pro-inflammatory state has been identified as an independent risk factor for functional decline, impaired mobility, and slow gait speed in older adults [4,5]. In principle, chronic inflammation is a modifiable risk factor, but it remains unclear whether attenuating inflammation in late life can truly improve or preserve physical function [6,7]. Recent trials targeting inflammation have yielded equivocal results [6,7]. Notably, the ENRGISE pilot trial (ENabling Reduction in low-Grade Inflammation in SEniors) tested whether anti-inflammatory interventions could improve mobility in older adults with high IL-6 and mobility impairment. ENRGISE randomized participants to the angiotensin receptor blocker losartan and/or omega-3 polyunsaturated fatty acids (fish oil) in a 2 × 2 factorial design. However, the interventions on average did not significantly reduce IL-6 or improve walking speed over 12 months [8]. These null average results highlight a critical gap: conventional approaches may overlook heterogeneity in responses among older individuals [9]. This is the central premise of precision geroscience—the idea that geriatric interventions might need to be tailored to an individual’s unique biology in order to be effective [10,11,12,13,14].

One emerging approach to enable precision geroscience is metabolomics, the analysis of low molecular weight compounds in biofluids or tissues [15,16]. Because metabolites serve as integrative readouts of genetic, proteomic, and environmental influences, metabolomics can sensitively reflect an individual’s physiological state [15,16]. In gerontology research, untargeted metabolomics has begun to reveal metabolic alterations associated with aging, chronic inflammation, and functional decline. For example, frail older adults exhibit distinct serum metabolite profiles (e.g., differences in energy metabolites and fatty acids) compared to non-frail peers [17]. Circulating metabolites have also been linked to physical performance measures such as gait speed and grip strength [18,19,20,21,22]. Importantly, certain metabolite signatures may predict future functional decline [23]. In the Health ABC cohort, higher levels of metabolites related to amino acid catabolism and kidney function at baseline were associated with a greater risk of developing mobility disability over 13 years [24]. These findings suggest that metabolic profiles could help identify individuals at risk of worsening function before clinical disability manifests. Such biomarkers could be invaluable for targeting interventions to those most likely to benefit.

Against this backdrop, integrating metabolomic profiling into clinical studies of aging may illuminate biologic heterogeneity and enable a precision geroscience paradigm [25]. Particularly in the context of the ENRGISE trial—where an intervention aimed at reducing inflammaging showed no mean benefit—metabolomics offers a means to probe underlying pathways and discover subgroups of “responders” or distinct aging phenotypes. Prior ENRGISE analyses have focused on traditional inflammatory biomarkers (e.g., IL-6) and clinical outcomes, but have not examined comprehensive metabolic changes [8,26]. We posit that untargeted metabolomics can uncover biochemical correlates of inflammaging and functional impairment, and potentially stratify older adults by their biological response to anti-inflammatory therapy.

In the present study, we conducted an untargeted metabolomics analysis of serum samples from ENRGISE participants to explore the interplay between systemic metabolism, inflammation, and functional aging. Our objectives were: (1) to characterize associations between baseline metabolomic profiles and physical function (muscle strength and gait speed) in older adults with chronic inflammation; (2) to determine whether baseline or longitudinal metabolomic patterns were related to trajectories of muscle strength or gait speed over 12 months; and (3) to evaluate the metabolic impact of the anti-inflammatory interventions (losartan and omega-3 supplementation) and identify metabolomic signatures of treatment response. We hypothesized that older adults with greater functional impairment at baseline would show distinct metabolic profiles reflecting pro-inflammatory and catabolic pathways, and that these baseline differences would predict differences in functional trajectories (improvement vs. decline). Furthermore, we anticipated that the interventions would induce specific shifts in metabolite pathways (e.g., lipid and eicosanoid metabolism) despite the lack of clinical improvement on average, consistent with biological activity in a subset of participants. By addressing these aims, this study seeks to fill critical gaps in our understanding of metabolomic underpinnings of inflammaging and functional decline, and to advance a precision geroscience approach for identifying biomarkers and therapeutic targets in vulnerable older populations.

## 2. Methods

### 2.1. Study Design and Participants

This study analyzed biospecimens and data from the ENRGISE Pilot randomized controlled trial, described in detail elsewhere [8,27]. In brief, ENRGISE was a multicenter, double-blind, placebo-controlled trial that enrolled community-dwelling older adults (≥70 years) with evidence of chronic inflammation and mobility limitation. Key inclusion criteria included: an elevated IL-6 level between 2.5 and 30 pg/mL at screening, self-reported or measured mobility impairment (but still able to complete a 400 m walk at baseline), and no severe disability. Participants were excluded for acute inflammatory conditions, recent major illness or surgery, or contraindications to the study medications. All participants provided written informed consent, and the protocol was approved by the institutional review boards of the participating institutions. A total of 289 participants were randomized between April 2016 and June 2017. Upon enrollment, baseline assessments included demographic and health questionnaires, a blood draw for biomarker analyses, and physical performance testing. Participants were then randomized in a 2 × 2 factorial design. Randomization was stratified by clinic site and baseline IL-6 level. This design yielded four intervention arms of roughly equal size: Losartan-only, Omega-3 only, Losartan + Omega-3 combination, and Placebo (placebo for both). The target sample size was 300 (75 per main effect group), and 289 were ultimately randomized, with slightly unequal group sizes due to stratification and dropouts.

### 2.2. Interventions and Follow-Up

Participants assigned to losartan received an initial dose of 25 mg per day, taken orally. After 1–2 weeks, the dose was increased to 50 mg/day if tolerated, with the goal of maintaining 50 mg daily through 6 months. Per protocol, if a participant’s average IL-6 at 3 and 6 months had not decreased by >40% from baseline (indicating insufficient inflammatory response), the losartan dose could be further increased to 100 mg/day from 6 to 12 months. Dose reductions or discontinuation were allowed for safety reasons (e.g., hypotension, hyperkalemia, renal function decline). Participants assigned to omega-3 supplementation received fish oil capsules (providing 1.4 g/day of fish oil, containing ~400 mg eicosapentaenoic acid [EPA] and 200 mg docosahexaenoic acid [DHA] per day). The fish oil dose was increased to 2.8 g/day at 6 months for participants whose IL-6 had not fallen by >40% at mid-point, unless contraindicated. Identical placebo capsules (containing inert corn oil for fish oil, and an inert pill for losartan) were used to maintain blinding. Participants and study personnel were blinded to assignment for the trial’s duration. The intervention period was 12 months. During this time, participants attended follow-up visits approximately every 3 months (at 3, 6, 9, and 12 months post-randomization). At each visit, adverse events and adherence were assessed, and physical function tests were repeated (schedule details below). Blood samples for metabolomic analysis were collected at baseline (pre-randomization), 6 months, and 12 months. These time-points were chosen to capture both mid-intervention and end-of-intervention metabolic profiles. Blood was drawn in the morning after an overnight fast when possible, processed to serum, and aliquoted for storage at −80 °C until batched metabolomic assays. The primary clinical endpoints of ENRGISE were IL-6 levels and mobility (400 m walk speed) at 12 months; however, for the present analysis, we focus on metabolomic and functional measures as described below.

### 2.3. Physical Function and Inflammation Measures

Physical function was assessed via grip strength and gait speed, which are standard, clinically relevant measures of muscle function and mobility in geriatric populations. Grip strength was measured using a hand dynamometer (JAMAR or similar), with participants instructed to squeeze maximally. Typically, three trials were performed per hand, and the maximum value (in kg) was recorded. We analyzed grip strength as the maximum dominant-hand grip. Usual gait speed was measured as the time to walk 400 m at a usual walking pace (the 400 m Walk Test). The 400 m walk was selected because all participants were able to complete this distance at baseline by the inclusion criteria. Grip strength and 400 m walk tests were conducted at baseline, 6 months, and 12 months, with additional interim short-distance walk tests at 3 and 9 months as part of the trial’s quarterly assessments. For certain analyses, we also considered walking speed over short distances (e.g., usual 4 m gait speed) and Short Physical Performance Battery (SPPB) scores, if available, to classify baseline mobility impairment; however, the primary mobility outcome was the 400 m walk speed.

Inflammatory markers: As part of trial screening and monitoring, plasma IL-6 was measured at baseline, 3, 6, and 12 months (by high-sensitivity ELISA) to confirm chronic inflammation and adjust doses. High-sensitivity C-reactive protein (CRP) was also measured at baseline. These inflammatory markers were used for participant selection and descriptive analyses; notably, all included individuals had IL-6 in the 2.5–30 pg/mL range at baseline by design. We examined whether baseline IL-6 levels (in quartiles) were associated with global metabolomic differences at baseline, as an indicator of “inflammaging” imprint on the metabolome.

Biospecimen Storage: Serum biospecimens were collected during the ENRGISE trial (final collection June 2017) and stored at the Aging Research Biobank at −80 °C until retrieval for this metabolomics project following the award of a Clin-STAR Pilot Grant (CS22005).

### 2.4. Metabolomics Sample Preparation

Metabolomics data was captured and processed (July–October 2023) in the Metabolomics and Exposome Laboratory (directed by Sumner) at the UNC Chapel Hill Nutrition Research Institute (UNC NRI) using methods and libraries developed under the NIEHS-funded Human Health Exposure Analysis Resource program (U2CES030857).

Serum samples were prepared for metabolomics analysis according to previously described methods. A volume of 50 µL of each serum sample was aliquoted for extraction. An additional 10 µL of each sample was combined to make a quality control study pool (QCSP), which was then distributed into 50 µL aliquots. National Institute of Standards and Technology (NIST) reference serum (SRM 909c) and LC-MS grade water were distributed into 50 µL aliquots to prepare external reference materials and method blanks, respectively. All quality control (QC) samples (QCSP, NIST, and blanks) were processed identically to the individual serum study samples.

Briefly, 4400 µL of an extraction solution (80% methanol and 20% water) with 500 ng/mL tryptophan-d5 as an internal standard was added to all samples, QCSPs, NISTs, and blanks. Samples were then vortexed for 2 min at 5000 rpm at room temperature, and centrifuged at 16,000× *g* for 10 min at 4 °C. Supernatants (350 µL) were transferred to new tubes and dried overnight by a SpeedVac (Labconco, Kansas, MO, USA). To reconstitute dried extracts, 100 µL of 95:5 water-methanol (*v*/*v*) was added to all samples, which were then vortexed for 10 min at 5000 rpm, and centrifuged at 16,000× *g* for 10 min at 4 °C. Supernatants of reconstituted extracts were then transferred into LC-MS autosampler vials, and 5 µL was used for the injection volume for all samples for untargeted analysis.

### 2.5. Ultra High Performance Liquid Chromatography-High Resolution Mass Spectrometry (UHPLC-HRMS) Data Acquisition

A Vanquish UHPLC connected to a Q Exactive™ HF-X Orbitrap Mass Spectrometer (Thermo Fisher Scientific, San Jose, CA, USA) was used to collect untargeted metabolomics data on all samples. An HSS T3 C18 column (2.1 × 100 mm2, 1.7 µm, Waters Corporation, Milford, MA, USA) held at 50 °C was used for the UHPLC column, and water (A) and methanol (B), both containing 0.1% formic acid (*v*/*v*), were used for the UHPLC solvents. The UHPLC gradient started at 2% B, was ramped up to 100% B over 16 min, and was then held at 100% B for 4 min. A flow rate of 400 µL/min was used throughout the UHPLC method.

Mass spectral data were acquired in positive ionization mode from 70 to 1050 *m*/*z*. A data-dependent acquisition (DDA) mode was used to collect MS/MS data on the top 20 most abundant ions per full scan. Injections of quality control samples (QCSPs, NIST reference materials, and blanks) were interspersed throughout the randomized study samples at a rate of 10% before the analysis.

A total of 736 study samples were analyzed across 9 analytical batches. After quality control, the final dataset included 731 serum study samples, with five samples removed due to injection errors (two baseline, two 6-month, and one 12-month sample).

### 2.6. Metabolomics Data Preprocessing

Raw instrument files were preprocessed using Progenesis QI (version 2.4, Waters Corporation, Milford, MA, USA) for feature extraction, alignment, and peak picking. Background signals were removed from the dataset by filtering out peaks with a higher average abundance in the blank samples as compared to the QCSP samples prior to normalization. All signals were then normalized to the total intensity within each sample, and batch effects were corrected. After normalization and batch effect correction, the coefficient of variation (CV) of all peaks was calculated across all QCSPs. Features with RSD > 50% were excluded from further analysis. Following this process, a total of 7050 peaks remained for downstream statistical analysis.

### 2.7. Compound Identification and Annotation

Metabolite identification/annotation was performed by matching signals to both an in-house RT, exact mass, MS/MS reference library and public mass spectral databases. The in-house library contained over 2400 metabolite standards at that time. Peaks were matched with accurate mass (±5 ppm), retention time (±0.5 min), and MS/MS spectral similarity (>30%). For public databases, peaks were matched to NIST, ToxCast, and HMDB databases, and matching was based on accurate mass (±5 ppm) and MS/MS spectral similarity (>30%).

A previously described ontology system was used to label each match according to the evidence supporting the metabolite assignment. OL1 represented the highest ontology level, indicating a match by exact mass (MS), MS/MS fragmentation patterns, and retention time (RT) to the in-house library. Features assigned as OL2a matched by exact MS and RT, while OL2b matched by MS and MS/MS spectra. For public database matches, PDa indicated a match by MS and experimental MS/MS spectra from NIST. PDb represented a match based on MS and theoretical MS/MS spectra from HMDB, while PDc included matches based on MS and isotopic similarity. Lastly, PDd indicated a match by MS alone. Due to the limitations of untargeted metabolomics, structural isomers, particularly those differing in D- and L-configurations, are not always distinguishable within the analytical platform used in this study. Matching of peaks to the in-house libraries and public databases, and automated assignment of ontology labels, was performed using ADAP-KDB (https://www.adap.cloud, accessed on 1 October 2023).

### 2.8. Statistical Analysis

The analysis plan addressed the three main objectives: baseline metabolomic associations, longitudinal trajectories, and intervention effects. Statistical analyses were performed in R 4.5.2 (R Foundation for Statistical Computing) and SAS 9.4 Software. We utilized the MetaboAnalystR package 4.0 for several analyses, including partial least squares discriminant analysis (PLS-DA) and pathway enrichment [28]. SAS PROC TRAJ was used for group-based trajectory modeling (GBTM) [29,30].

We also explored baseline metabolomic differences by sex and by race, independent of physical function. We tested for associations between baseline inflammatory level and the metabolome: participants were stratified into IL-6 quartiles, and we used one-way ANOVA and PLS-DA to see if baseline metabolomic profiles differed across three low to high IL-6 groups (1st quartile, 2nd and 3rd quartiles combined, and 4th quartile).

Cross Sectional Analysis: We first examined cross-sectional associations at baseline between metabolomic profiles and sex, race, IL-6 quartiles, grip strength and walking speed groups that were defined as follows. For IL-6, participants were categorized into quartiles and grouped into three ordinal IL-6 categories (lowest quartile, combined 2nd–3rd quartiles, and highest quartile), and we used one-way ANOVA and PLS-DA to assess whether baseline metabolomic profiles differed across these IL-6 groups. Grip strength has well-established sex differences and cut-points; therefore, we defined low muscle strength (weakness) based on sex-specific cutoffs (~<27 kg for men and <16 kg for women) as suggested by consensus guidelines [31]. Participants were classified as “weak” vs. “normal” strength, and metabolomic differences between these groups were assessed. Similarly, for gait, we defined slow walking speed using a cutoff of 0.8 m/s for usual gait (a common threshold for mobility impairment), and compared metabolomic profiles between slower vs. faster walkers at baseline. For each binary comparison, we performed univariate tests on each metabolite feature (two-sample *t*-tests on log-intensity values) to identify significantly different features (*p* < 0.05, with false discovery rate (FDR) correction applied as appropriate given the large number of features). Results were visualized in volcano plots. In addition, we conducted a supervised multivariate PLS-DA to see if the overall metabolomic profile could distinguish, for example, weak vs. non-weak participants. Model quality was evaluated via permutation testing and goodness-of-fit (R^2^) and prediction (Q^2^) statistics.

Grip Strength and Walking Speed Trajectories: To investigate how baseline and longitudinal metabolomic patterns relate to functional changes over time, we derived trajectory groups for change in grip strength and gait speed using GBTM. GBTM fits a semiparametric mixture model to longitudinal data with the use of the maximum-likelihood method. The SAS TRAJ procedure calculates the probability of each subject belonging to each trajectory group and assigns each subject to the group with the largest probability. The final model was selected based on optimizing the Bayesian Information Criterion (BIC). Additional criteria for model selection required that the smallest trajectory group size was at least 10% of the total sample, and posterior probabilities for group membership had to be >80%. We identified distinct subsets of participants who exhibited improvement, maintenance, or decline in grip strength and walking speed over 12 months. We then assessed baseline metabolomic differences among these trajectory groups using ANOVA and pairwise comparisons, to identify metabolites whose baseline levels differed in those who would go on to improve vs. decline (etc.). A one-way ANOVA was conducted across the three groups for each feature, and additionally, PLS-DA was used (with three-class discrimination) to evaluate multivariate separation. To examine whether longitudinal metabolomics profiles in the ENRGISE Pilot Study dataset relate to functional changes over time, we used data from baseline and two follow-ups (6 and 12 months). For each metabolic feature, we generated time-integrated concentrations (TICs) calculated as the area under the curve defined by the individual values of the corresponding metabolite at baseline, 6 months, and 12 months. Participants with missing data at least at one time point were excluded. Therefore, each participant with complete data at the baseline and two follow-ups had a set of TICs (one for each metabolite), which represents each participant’s longitudinal metabolomic profile. All longitudinal analyses were performed using the original continuous metabolite intensity values (semi-quantitative abundances), with log-transformation applied as needed to meet model assumptions. Next, we analyzed the TICs in relation to trajectories. Significant metabolites that differed by trajectory (either at baseline or in change over time) were compiled for pathway enrichment analysis. We performed analogous analyses for 400 m walk time trajectories and noted any consistent findings.

Intervention Effects on Longitudinal Metabolomic Profiles: To evaluate the biochemical impact of the interventions, we compared longitudinal metabolomic profiles represented by TICs between treatment arms using the MetaboAnalystR package as described above. PLS-DA models were constructed to visualize metabolomic separation between treatment groups (for instance, a PLS-DA of Omega-3 vs. Placebo at 12 months). VIP (Variable Importance in Projection) scores from these models helped identify the top discriminatory metabolic features.

Pathway Enrichment Analysis: To interpret the biological implications of metabolomic differences, we performed metabolic pathway enrichment analysis on sets of significant features from the above comparisons. Pathway enrichment analysis provides a means to highlight biological significance using features detected in the untargeted analysis, and does not require prior identification and annotation of the peaks. comwas conducted using the mummichog algorithm in MetaboAnalystR. We input the lists of significant *m*/*z* features to identify pathways that were significantly perturbed between the phenotypic group comparisons. Prior to all statistical analyses, metabolite intensities were normalized and corrected for inter-batch variation using batch-effect correction procedures. Enrichment *p*-values were calculated by permutation. We conducted pathway analysis for several key contrasts: (a) baseline low grip strength vs. normal, (b) baseline slow walk speed vs. normal, (c) baseline trajectory groups (improve/decline), and (d) each intervention effect (e.g., metabolites changed by omega-3). This allowed us to move beyond individual unknown metabolites to a higher-level understanding of which biochemical pathways (e.g., amino acid metabolism, lipid signaling, etc.) were most perturbed in relation to physical function or treatment.

All significance tests were two-tailed with an alpha of 0.05, and where applicable (feature-level analyses), we report FDR-adjusted q-values. Data are presented as mean ± standard deviation for normally distributed variables, or median (IQR) for skewed variables. Analyses were performed on the per-protocol population with available samples; sensitivity analyses on imputed missing data or the intent-to-treat sample did not appreciably differ and are not shown. Results are reported in accordance with STROBE guidelines for observational analyses of RCT data.

## 3. Results

After filtering and pre-processing of the untargeted metabolomics data, 7050 peaks were available for downstream analysis, of which 5633 were identified or annotated to the in-house physical standards library or public databases (NIST, HMDB, ToxCast). The unsupervised PCA showed that the 73 QC study pools clustered in the center of the study samples from which they were derived, which is a common practice to assess untargeted metabolomics data.

### 3.1. Baseline Metabolomic Profiles and Baseline Characteristics of the Cohort

Sex-based differences in metabolomic profiles were observed, with multiple lipid species and amino acids showing higher levels in men compared to women (Supplemental Figure S1, Supplementary Table). Racial differences were also present, with specific bile acid and lipid metabolites differing between Black and white participants (Supplemental Figure S2, Supplemental Table).

### 3.2. Baseline Metabolomic Profiles, Chronic Inflammation and Physical Function

**Figure 1:**
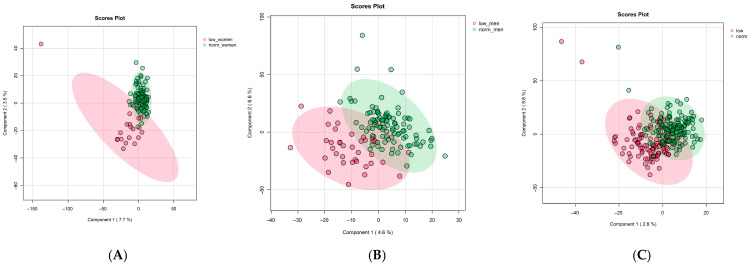
Partial Least Squares-Determinant Analysis (PLS-DA) plot demonstrating (**A**) the separation of females with low (<16 kg) vs. normal (≥16 kg) muscle strength at baseline using supervised multivariate analysis; (**B**) the separation of males with low (<27 kg) vs. normal (≥27) muscle strength at baseline; (**C**) the separation of participants with slow walking speed (<0.8 m/s) vs. normal walking speed (>0.8 m/s). Component 1 and Component 2 denote the first and second components, respectively; the numbers in parentheses indicate the percentage of the variance explained. Q2 was based on a 5-fold cross validation.

**Figure 2:**
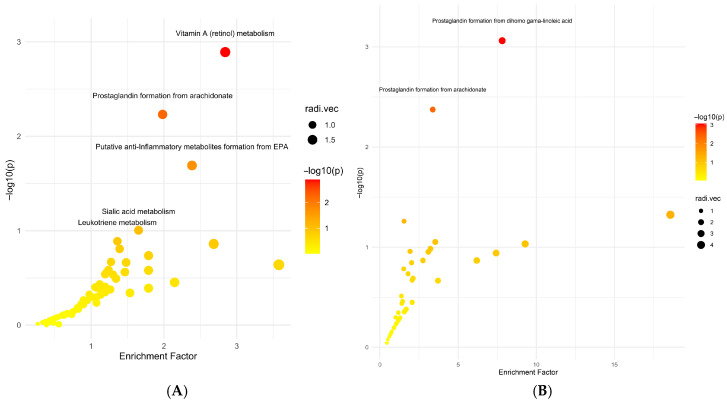
Metabolite pathway enrichment. *p*-values and fold changes were calculated for females with low muscle strength vs. females with normal muscle strength at baseline (**A**) and female and male participants with slow walking speed vs. participants with normal walking speed at baseline (**B**) for 7050 peaks. A *p*-value cutoff of 0.1 was used for the mummichog algorithm.

No significant associations were observed between baseline IL-6 levels and the metabolomics profiles (Supplemental Figure S3). Metabolomic profiles did not differ across quartiles of IL-6 (*p* > 0.1 for all features), and no clustering by IL-6 levels was detected in multivariate analyses. In contrast, physical performance measures were associated with distinct metabolomic signatures (Figure 1). Participants classified as having low muscle strength based on sex-specific grip strength cutoffs exhibited metabolic differences compared to those with normal strength (~80% women and ~65% men were accurately classified, but a negative Q2 indicates reproducibility is unlikely. A total of 609 peaks with *p* < 0.5 (comparing females with low muscle strength < 16 kg vs. normal muscle strength ≥ 16 kg at baseline) were analyzed using the Mummichog module in the MetaboAnalystR package. Pathway enrichment analysis (Figure 2A) identified significant enrichment for the Vitamin A (retinol) metabolism pathway (Fisher’s Exact Test *p* = 0.001; 13 metabolites), the Prostaglandin formation from arachidonate pathway (*p* = 0.006; 15 metabolites), and the putative anti-inflammatory metabolites formation from EPA pathway (*p* = 0.02; 8 metabolites). After FDR correction (threshold *p* < 0.1), only the Vitamin A (retinol) metabolism pathway remained significant (*p*.adj = 0.08).

The PLSDA visualization for the participants with slow walking speed (<0.8 m/s) vs. normal walking speed (>0.8 m/s) showed overlap between the two groups, and the Q2 statistic for this model was negative (Q2 = −0.1, Figure 1C). However, 60% were accurately classified when using Xi components. In pathway enrichment analysis, participants in the lowest quartile of 400 m walking speed (<0.8 m/s) showed differences in pathways related to inflammation and amino acid metabolism (Figure 2B). Prostaglandin and eicosanoid biosynthesis pathways were more active in slower walkers, with multiple metabolite features putatively linked to prostaglandin production showing higher relative abundance in this group. Features associated with tyrosine metabolism were also significantly altered in slow walkers.

### 3.3. Baseline Metabolomics and Trajectories in Grip Strength and Walking Speed

**Figure 3:**
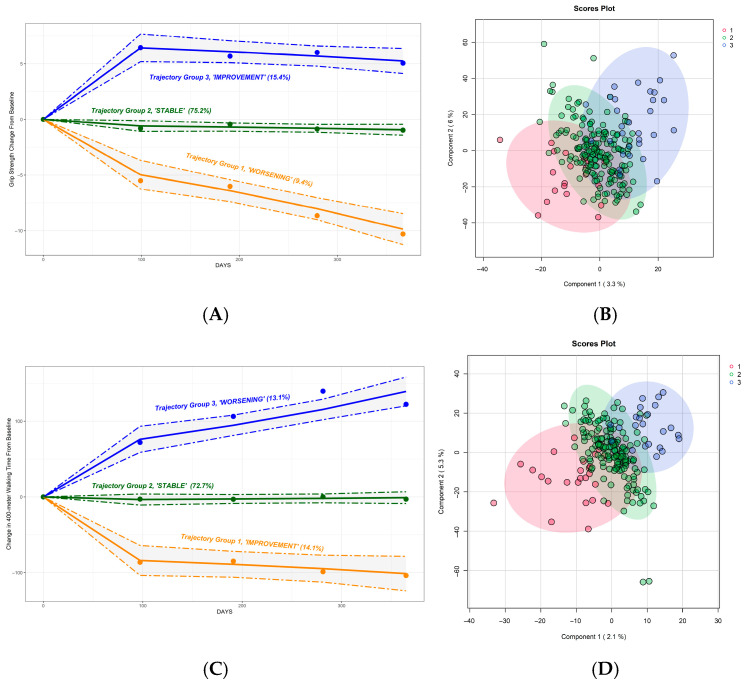
Baseline Metabolomics Differentiates Grip Strength and Walking Speed Trajectories. (**A**) Grip strength trajectories identified by group-based trajectory modeling (N = 289). (**B**) Baseline metabolomics by grip strength trajectories (N = 229, 60 participants with missing metabolomics data and/or uncertain assignment to the trajectory group were excluded); the groups are colored by the trajectory group corresponding to Figure 3A (blue: declined [N = 20], green: stable [N = 170] remained stable, red: improved grip strength [N = 30]). (**C**) Walking speed trajectories identified by group-based trajectory modeling (N = 289). (**D**) Baseline metabolomics by walking speed trajectories (N = 213, 76 participants with missing metabolomics data and/or uncertain assignment to the trajectory group were excluded); the groups are colored by the trajectory group corresponding to Figure 3A (blue: declined [N = 23], green: stable [N = 165] remained stable, red: improved walking speed [N = 25]).

Three distinct trajectory groups were identified for each measure (Figure 3A,C). To ensure classification certainty, participants with posterior probabilities below 0.8 were excluded (walking speed trajectories: 53 participants excluded, grip strength trajectories: 32 participants excluded). For grip strength, 22 of the participants experienced a decline, 203 remained stable, and 32 showed improvement (Figure 3A). For walking speed, 26 participants declined, 183 remained stable, and 27 improved. Table 1 and Table 2 summarize baseline characteristics for participants in each trajectory group following the exclusion of individuals with posterior probabilities below 0.8.

In total, 257 participants with grip strength trajectory assignments had baseline metabolomics data available for the analysis. Baseline metabolomic profiles were associated with trajectories of grip strength and walking speed over 12 months (Figure 3B). In grip strength trajectory analyses, PLS-DA score plots revealed clustering of individuals based on their future functional trajectory, with separation observed between those who declined (N = 20), remained stable (N = 179), or improved (N = 30) (Figure 3B, top panel). Among 236 participants with walking speed trajectory assignments, 213 had baseline metabolomics data (23 declined, 165 remained stable, 25 improved walking speed). Similarly, for walking speed trajectories, metabolomic differences were evident at baseline, with PLS-DA analysis showing partial separation between functional groups (Figure 3D, bottom panel). Despite differentiation, no specific pathways were identified as significantly different between trajectory groups in pathway analysis.

### 3.4. Longitudinal Metabolomics and Trajectories in Grip Strength and Walking Speed

**Figure 4:**
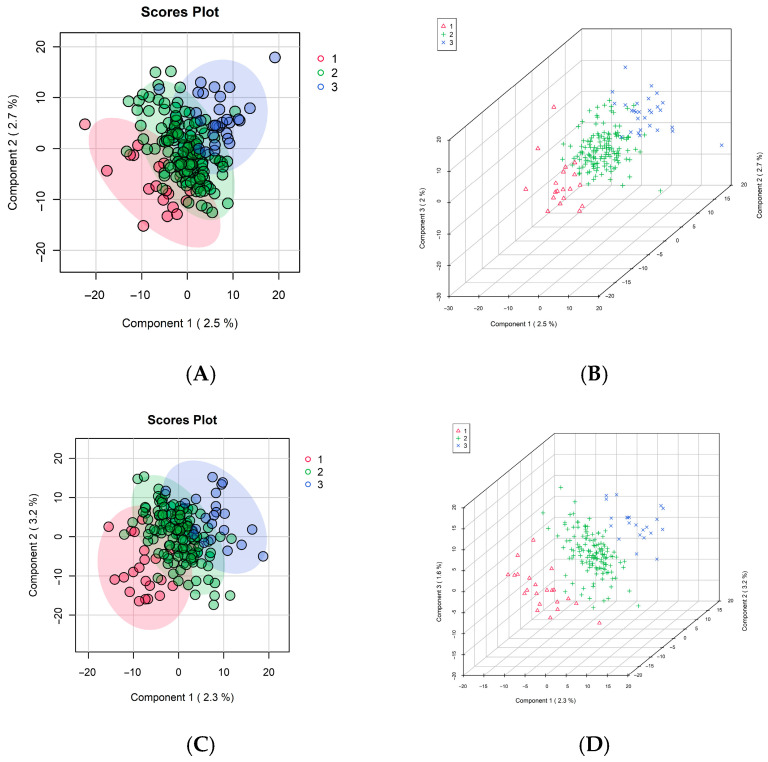
Untargeted Longitudinal Metabolomics Differed Across Grip Strength and Walking Speed Trajectories. (**A**,**B**) Longitudinal metabolomics and grip strength trajectories. (**C**,**D**) Longitudinal metabolomics and walking speed trajectories.

Longitudinal metabolomic profiling revealed distinct metabolic changes associated with grip strength and walking speed trajectories over 12 months (Figure 4). Multivariate analysis demonstrated separation of these groups based on changes in metabolomic profiles over time. In grip strength trajectory analyses, a supervised PLS-DA model identified distinct clustering patterns among the three groups, as shown in Figure 4A,B. The 2D and 3D score plots indicate separation between individuals with declining, stable, and improving grip strength, suggesting that specific metabolic shifts were associated with functional changes. However, again, despite differentiation, no specific pathways were identified as significantly different between trajectory groups in pathway analysis of longitudinal metabolomics.

### 3.5. Intervention Effects on Longitudinal Metabolomic Profiles

**Figure 5:**
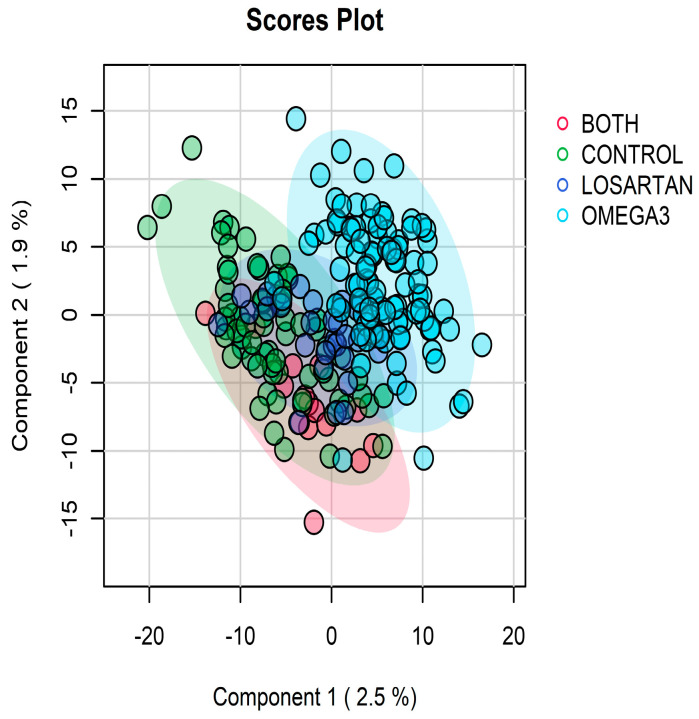
Untargeted longitudinal metabolomics data distinguished participants from different treatment groups.

Longitudinal metabolomic profiling identified distinct metabolic signatures associated with treatment group allocation. A total of 210 participants with complete longitudinal data were included in this analysis. Supervised PLS-DA analysis revealed separation between participants receiving omega-3 supplementation alone (N = 96), those receiving losartan plus omega-3 (N = 15), and those in the control group (N = 71) (Figure 5, left panel). To enhance the analysis, we further compared the omega-3 group versus the control group and the losartan plus omega-3 group versus the control. In the omega-3 group, pathway enrichment analysis using the mummichog algorithm identified several significantly perturbed metabolic pathways. These included “de novo fatty acid biosynthesis,” “fatty acid activation,” “omega-3 fatty acid metabolism,” “D4&E4-neuroprostanes formation,” and “prostaglandin formation from dihomo-gamma-linolenic acid. Tyrosine metabolism were also significantly altered in slow walkers.

In the losartan plus omega-3 group, significant pathway perturbations were also observed, including “fatty acid activation,” “omega-3 fatty acid metabolism,” “D4&E4-neuroprostanes formation,” and “prostaglandin formation from dihomo-gamma-linolenic acid,” similar to the omega-3 group. Additionally, this group exhibited changes in “pyrimidine metabolism”. Pathway enrichment analysis was performed using the Mummichog algorithm, applying a significance threshold of *p* < 0.05 to select features for inclusion. This threshold was chosen to reduce false positives while comparing metabolic effects across randomized treatment arms (omega-3 supplementation, losartan plus omega-3, and control).

## 4. Discussion

In this study, we leveraged untargeted metabolomics to investigate the biological underpinnings of aging, chronic inflammation, and functional decline in a cohort of older adults participating in an anti-inflammatory intervention trial. Several key findings emerged. First, we identified distinct metabolomic profiles associated with baseline physical function, with older adults exhibiting weaker grip strength and slower gait displaying metabolic signatures enriched in vitamin A and inflammatory lipid pathways compared to their better-functioning peers. Second, baseline metabolomic patterns were associated with future functional trajectories, suggesting that metabolic phenotypes may predispose individuals to functional decline or stability over 12 months. Third, although the interventions (losartan and omega-3 fish oil) did not improve physical function on average, they induced specific metabolic alterations consistent with their expected mechanisms. Omega-3 supplementation significantly impacted fatty acid and eicosanoid metabolism, confirming pharmacologic activity, whereas losartan’s metabolic effects were minimal.

### 4.1. Baseline Metabolomic Signatures of Low Function

The cross-sectional associations observed in this study align with and extend prior research on metabolic biomarkers of frailty and disability. For example, Pan et al. identified several serum metabolites differentiating frail vs. non-frail older adults, including intermediates of carbohydrate metabolism and fatty acids, and reported multiple metabolites correlated with grip strength and gait speed [17]. Our finding that vitamin A (retinol) metabolism was the most perturbed pathway in women with low grip strength is particularly notable. Vitamin A and its active derivatives (retinoic acids) play essential roles in muscle protein synthesis and regeneration [32,33]. Both deficiency and excess of retinoids have been implicated in muscle dysfunction [32]. Recent studies have linked retinol-binding protein 4 (RBP4)—a key transporter of vitamin A—to sarcopenia and frailty [34]. Elevated circulating retinol and RBP4 have been associated with lower muscle mass and strength in older adults, possibly through mechanisms involving insulin resistance and inflammation [34]. The disruption of vitamin A pathways in participants with weak grip strength in our study supports emerging evidence that retinoid dysregulation may contribute to sarcopenia [33]. Similarly, the enrichment of prostaglandin and eicosanoid pathways in individuals with slower gait speed aligns with the hypothesis that pro-inflammatory lipid mediators contribute to mobility impairment [35]. Prostaglandin E2 has been shown to promote muscle catabolism and pain, which could contribute to reduced physical activity [35,36]. Additionally, observed differences in tyrosine metabolism in slow walkers may indicate altered neurotransmitter synthesis (dopamine, epinephrine) affecting motor function, or they may reflect metabolic stress, as the accumulation of tyrosine byproducts is associated with mitochondrial inefficiency [37]. Interestingly, we found no significant association between baseline IL-6 levels and the untargeted metabolomics profile in this cohort. Given IL-6’s role in systemic inflammation, one might expect that higher IL-6 levels would correlate with a more pro-inflammatory metabolome. The absence of this association may be due to the restricted IL-6 range in our cohort (as all participants had IL-6 > 2.5 pg/mL, limiting contrast), or it may reflect the fact that IL-6 alone does not fully capture the complexity of inflammatory metabolic processes. These findings highlight the value of untargeted metabolomics in assessing inflammaging, as single cytokine biomarkers like IL-6 may be insufficient for stratifying biological risk. Instead, a composite metabolomic profile may provide a more comprehensive assessment of inflammation-related metabolic dysregulation.

### 4.2. Metabolomics and Functional Trajectories

A novel contribution of our study is the demonstration that baseline metabolomic differences foreshadowed divergent functional outcomes. We found that participants who maintained or improved their grip strength had a baseline metabolic profile distinct from those who declined, even though their initial clinical measures were similar. We also observed that metabolomic changes over time mirrored functional changes. This dynamic coupling implies potential mechanistic links. It is worth noting that despite the ability to differentiate groups using baseline metabolomics and longitudinal metabolomics, pathway analysis did not identify any significant pathways that were associated with grip strength or gait speed groups. Therefore, these observations should be viewed as hypothesis-generating. These results suggest that metabolomic biomarkers could serve as early indicators of resilience or vulnerability in older individuals. Prior longitudinal studies offer supporting evidence: in the Health ABC study, as noted earlier, higher levels of certain toxin-related metabolites (indoxyl sulfate, symmetric dimethylarginine) predicted greater risk of mobility disability over 10+ years, indicating a less healthy metabolic state predisposes to decline [24]. These findings further raise the question: are the metabolite changes merely biomarkers of the functional change, or do they play a causal role in driving the change? It is possible that certain metabolic states (e.g., chronic amino acid catabolism, energy deficit) actively contribute to muscle loss. If so, metabolites could be targets for interventions—for example, reducing toxic metabolites like indoxyl sulfate (perhaps via gut microbiome modulation) or supplementing deficient metabolites—to help prevent decline. Future studies should test whether modifying these metabolic pathways can influence physical outcomes. Our results contribute to the paradigm of using *omics* to define phenotypic trajectories in aging. The concept of “precision geroscience” emphasizes that individuals vary greatly in their biological aging processes. Tools like metabolomics allow us to quantify this variation. In our study, even though all participants were fairly similar in chronological age and all had chronic inflammation by selection, their metabolomes distinguished them into subgroups with different futures. This supports the notion that chronological age is not the sole driver—biologic age or health status, captured by metabolomic (and likely other ‘omic’) profiles, is what influences functional decline. Metabolomics could thus be incorporated into geriatric assessments to improve risk stratification beyond conventional clinical metrics.

### 4.3. Interpreting Intervention Effects

The ENRGISE trial reported no significant benefits of losartan or fish oil on its primary outcomes (IL-6 and 400 m walk). Our metabolomic analysis adds an important layer to that outcome by confirming the interventions did have the expected biochemical effects, especially for fish oil. This finding has two implications. First, it validates that the lack of functional improvement was not simply due to non-adherence or inadequate dosing of fish oil—the metabolomic changes (increased omega-3 lipid metabolites, etc.) show that the supplement was indeed taken and incorporated in the majority of participants. The fish oil led to changes in pathways associated with de novo fatty acid biosynthesis,” “fatty acid activation,” “omega-3 fatty acid metabolism,” “D4&E4-neuroprostanes formation,” and “other studies of omega-3 in older adults, and created an anti-inflammatory lipid milieu (e.g., prostaglandin formation from dihomo-gamma-linolenic acid) [38]. Yet, this was insufficient to translate into better gait speed or strength in 12 months. This suggests that simply reducing systemic inflammation (via fish oil’s mechanism) may not overcome the multi-factorial causes of mobility loss in this population, or that a longer duration is needed to see functional effects. Second, the metabolomic results highlight the difference in mechanism between the two interventions: omega-3 primarily affects metabolic substrates and inflammation, while losartan—an ARB—did not significantly alter circulating metabolites. Losartan’s benefits, if any, would come through improved hemodynamics, reduced angiotensin II signaling, and perhaps muscle perfusion, none of which produced a strong metabolic fingerprint in blood in this pilot. The minimal metabolomic effect of losartan could also be due to the dosing issues (many participants could not be titrated to a high dose or stopped early). Thus, it is plausible that an effectively dosed ARB might show metabolic effects (for example, improved mitochondrial efficiency or insulin sensitivity reported with ARBs in some studies), but we did not observe that here. One novel observation was the alteration in pyrimidine metabolism in the combination therapy group. Why might combining losartan with omega-3 affect nucleotide metabolism? One hypothesis is that improved blood pressure (losartan) plus anti-inflammatory effects (omega-3) might together reduce cellular stress, leading to changes in nucleotide turnover or salvage pathways. Alternatively, it could be a chance finding in a small subgroup, or related to unmeasured factors. Regardless, it raises an interesting question for future research: do geroprotective strategies (combining interventions) tap into metabolic pathways beyond those targeted by single agents? If yes, metabolomics can help discover these unexpected pathway modifications.

### 4.4. Implications for Precision Geroscience and Future Directions

Our findings underscore the importance of biological heterogeneity in aging and intervention responses. While the trial’s overall effects on function were null, metabolomic data revealed clear biological engagement of omega-3, with altered fatty acid and inflammatory lipid metabolism. This suggests that responses to interventions may vary by baseline metabolic profile. Future work should explore whether individuals with specific pre-treatment signatures (e.g., low omega-3 index, high inflammatory metabolites) are more likely to benefit. Identifying such metabolic subtypes could advance precision geroscience, enabling targeted interventions for those most likely to respond. Despite no observed clinical benefit, omega-3 supplementation shifted metabolism in a direction generally considered favorable (e.g., reduced inflammatory lipids, altered eicosanoid pathways). This raises important questions: Are standard functional endpoints too crude to detect meaningful improvements? Would longer follow-up or multimodal interventions (e.g., omega-3 plus exercise or an anti-inflammatory agent) yield greater benefits? Given that aging involves multiple pathways, interventions targeting multiple hallmarks of aging may be necessary to translate metabolic changes into functional gains. To build on these findings, future studies should validate key metabolic associations in larger cohorts and test whether specific metabolomic profiles predict functional trajectories. Targeted quantification of metabolites identified here (e.g., retinol, RBP4, prostaglandins, 3-methylhistidine) could determine their utility as biomarkers for identifying individuals at risk of decline. Mechanistic studies are also warranted to clarify whether modifying these pathways (e.g., through retinoid signaling, inflammatory lipid modulation, or mitochondrial support) influences physical function. Ultimately, this work contributes to efforts to develop metabolomic biomarkers for aging and frailty. A validated panel of circulating metabolites could help stratify older adults by biological risk and guide personalized interventions. While clinical implementation is still distant, studies like ours offer proof-of-concept for integrating metabolomics into geroscience trials to refine interventions and optimize healthspan.

### 4.5. Limitations

This study has several limitations. First, the sample size, particularly for subgroup analyses (trajectory groups, treatment subgroups), was modest. This limits statistical power to detect smaller effects and to perform extensive multivariable adjustments. PLS-DA was used to explore potential group separation; however, all Q2 values were negative, and the variances explained by PC1 and PC2 were low, suggesting that the models have limited predictive ability and may be less likely to be reproducible in an independent sample. This indicates that the observed patterns should be interpreted cautiously and considered exploratory rather than confirmatory and in need of replication. Second, the untargeted metabolomics approach, while broad, measured a subset of the metabolome (only those detectable in positive ion mode in serum). As no single analytical method can detect the entire metabolome, additional analyses using negative ion mode, other analytical platforms and other biospecimen types can provide a more comprehensive picture of the metabolome. Additionally, as is the case with the majority of untargeted metabolomics studies, many of the features detected remain unidentified, which limits biological interpretation. We relied on pathway enrichment (which focused on host metabolism) for biological interpretation. Specific causal relationships (e.g, influence of environmental analytes) have not been evaluated and will be presented in future papers. Third, multiple hypothesis testing is inherent in metabolomic analyses; we mitigated this by focusing on pathway-level inferences and consistent patterns rather than individual metabolite *p*-values, but the risk of type I errors remains. Fourth, our trajectory classification for grip strength was done post hoc and may not generalize—different trajectory cutoffs might yield different results. We also did not account for all factors that influence muscle strength (like intercurrent illnesses or exercise during the year) which could confound the trajectory-metabolite associations. Fifth, regarding interventions, because this was a secondary analysis of a trial, participants were not randomized by metabolomic profile. Thus, any interactions we postulate between baseline metabolism and treatment response are observational and need confirmation in a prospective manner.

Another limitation is that the ENRGISE pilot was relatively short (1 year) and focused on a specific high-risk population. The metabolomic associations we found might differ in other populations (for instance, healthier older adults might have different key pathways, or advanced frail elders might have others). Also, the interventions were specific (ARB and fish oil); other anti-inflammatory or pro-muscle interventions (e.g., IL-6 blockers, amino acid supplements, exercise) could have very different metabolomic effects. Our results are specific to the combination of chronic inflammation and mobility impairment context. Finally, we acknowledge that correlation does not imply causation—while we see metabolomic changes alongside functional changes, we cannot be sure if one causes the other. It is possible that an unmeasured factor (like a change in diet or physical activity) influenced both metabolism and function in parallel. However, the randomized design for the intervention analysis does strengthen the inference that the observed omega-3 metabolite changes were caused by the treatment.

## 5. Conclusions

In this cohort of mobility-impaired older adults with chronic inflammation, untargeted metabolomic profiling revealed associations between baseline performance measures and physical function trajectories. Baseline metabolomic profiles differed by functional status and predicted future trajectories of grip strength and walking speed. Additionally, omega-3 supplementation produced expected metabolic shifts, particularly in fatty acid and eicosanoid metabolism, despite no observed clinical improvements in function. These findings demonstrate the potential of metabolomics to provide a biological context for functional decline and intervention responses in aging populations. Future research should focus on validating these metabolic signatures in larger cohorts and exploring their role in identifying individuals who may benefit from targeted interventions. By integrating multi-omics approaches into geriatric trials, we can improve risk stratification and intervention design to better support healthy aging.

## Figures and Tables

**Table 1:** Baseline Characteristics by Grip Strength Trajectories.

Characteristic	Worsening Group (N = 22)	Stable Group (N = 203)	Improvement Group (N = 32)	*p*-Value
Grip Strength, mean	38.2 (7.2)	25.3 (8.1)	23.9 (8)	<0.001
Age, years	75.6 (4.7)	77.7 (5.4)	76.2 (4.7)	0.11
Female, n (%)	4 (18.2%)	115 (56.7%)	7 (21.9%)	<0.0001
Anemia, n (%)	0 (0%)	35 (17.3%)	2 (6.3%)	0.03
Cancer, n (%)	9 (40.9%)	72 (35.5%)	13 (40.6%)	0.78
Depression, n (%)	3 (13.6%)	40 (19.7%)	5 (15.6%)	0.70
Elevated Wait Circumference, n (%)	19 (90.5%)	166 (82.6%)	22 (71%)	0.17
High BP, n (%)	18 (81.8%)	134 (66%)	23 (71.9%)	0.28
Hypercholesteremia, n (%)	15 (68.2%)	122 (60.1%)	19 (59.4%)	0.75
IL6, mean	4.1 (1.5)	4.7 (3)	4.9 (4)	0.65
Metabolic Syndrome Score, mean	2.8 (1.1)	2.3 (1)	2.3 (1)	0.14
Myocardial Infarction, n (%)	2 (9.1%)	19 (9.4%)	1 (3.1%)	0.50
Mini Mental Status Exam Score, mean	27.9 (1.7)	28.1 (1.7)	28 (1.8)	0.89
Osteoarthritis, n (%)	12 (54.5%)	85 (42.1%)	12 (37.5%)	0.44
Stroke, n (%)	1 (4.5%)	9 (4.4%)	2 (6.3%)	0.90
Type II DM, n (%)	9 (40.9%)	44 (21.7%)	7 (21.9%)	0.13

**Table 2:** Baseline Characteristics by Walking Speed Trajectories.

Characteristic	Improvement Group (N = 27)	Stable Group (N = 183)	Worsening Group (N = 26)	*p*-Value
400 M Walk Time, mean	632.6 (134.3)	470.2 (89.8)	504 (83.6)	<0.001
Age, years	77.3 (4.4)	77.2 (5.4)	78.9 (5.9)	0.31
Female, n (%)	9 (33.3%)	86 (47%)	13 (50%)	0.37
Anemia, n (%)	4 (14.8%)	26 (14.2%)	5 (20%)	0.75
Cancer, n (%)	8 (29.6%)	68 (37.2%)	10 (38.5%)	0.73
Depression, n (%)	5 (18.5%)	37 (20.2%)	8 (30.8%)	0.44
Elevated Waist Circumference, n (%)	18 (66.7%)	152 (84.4%)	19 (73.1%)	0.05
High BP, n (%)	16 (59.3%)	132 (72.1%)	16 (61.5%)	0.26
Hypercholesteremia, n (%)	18 (66.7%)	114 (62.3%)	13 (50%)	0.41
IL6, mean	3.9 (1.1)	4.9 (3.3)	4.2 (2.2)	0.20
Metabolic Syndrome Score, mean	2.2 (1.2)	2.4 (1)	2 (0.9)	0.16
Myocardial Infarction, n (%)	3 (11.1%)	13 (7.1%)	6 (23.1%)	0.03
Mini Mental Status Exam Score, mean	28.1 (1.8)	28 (1.7)	28.1 (1.7)	0.90
Osteoarthritis, n (%)	13 (48.1%)	76 (41.8%)	15 (57.7%)	0.28
Stroke, n (%)	1 (3.7%)	9 (4.9%)	2 (7.7%)	0.79

## Data Availability

The original contributions presented in this study are included in the article/Supplementary Material. Further inquiries can be directed to the corresponding author.

## Supplementary Materials

The following supporting information can be downloaded at: https://www.mdpi.com/article/10.3390/metabo16010009/s1, Figure S1: Baseline metabolomics by Sex, Figure S2: Baseline metabolomics by Race. Figure S3: Baseline metabolomics by IL-6 quartiles, Supplementary Table: *t*-test statistics, fold-changes, and VIP values.
