# Supplementary material for: Metabolomic Signatures of Physical Function and Functional Trajectories in Older Adults: Insights from the ENRGISE Clinical Trial
Source: Metabolites. 2025 Dec 22;16(1):9. doi: 10.3390/metabo16010009 (PMC12844133; doi:10.3390/metabo16010009)

Supplemental Figure S1.

Differences by gender (baseline)

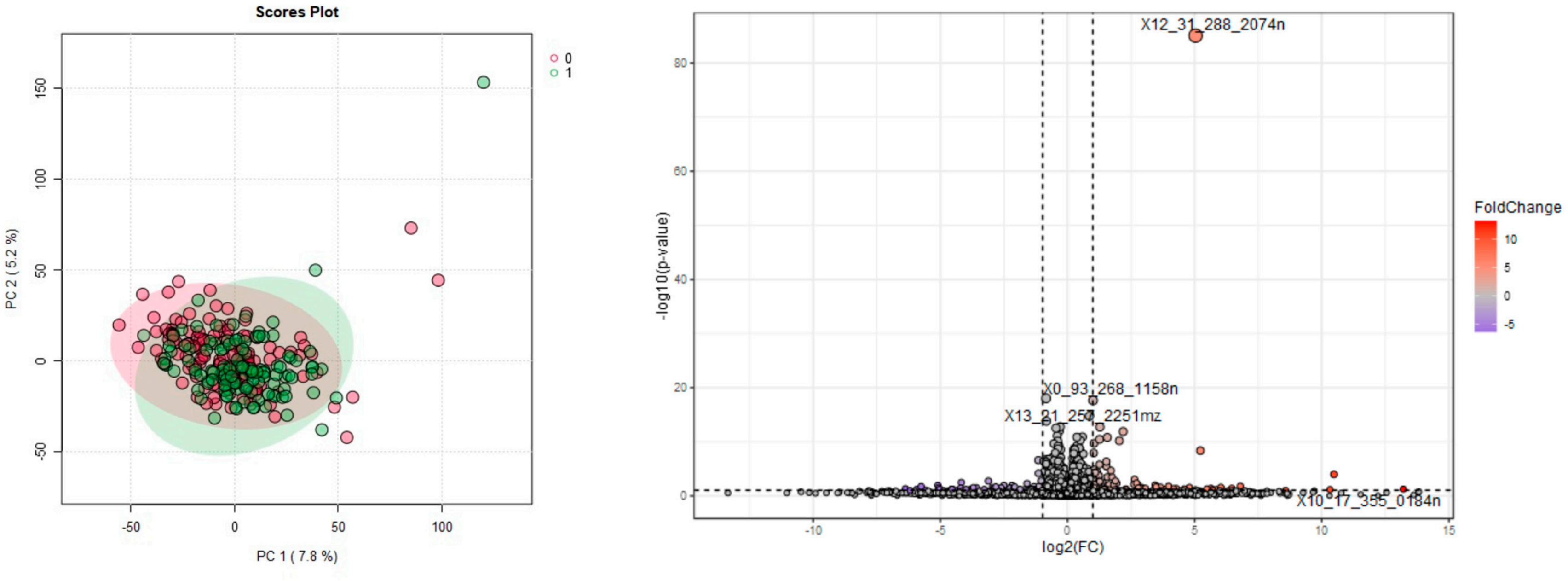

Supplemental Figure S2.

Differences by race (baseline metabolomics data) –volcano and pa[illegible]

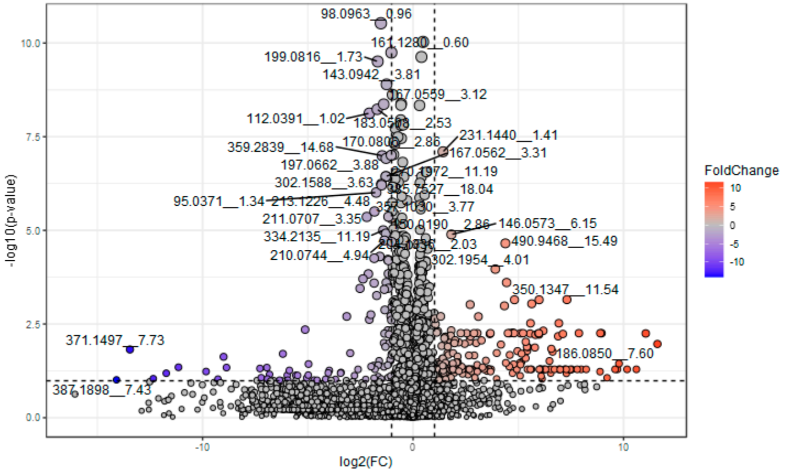

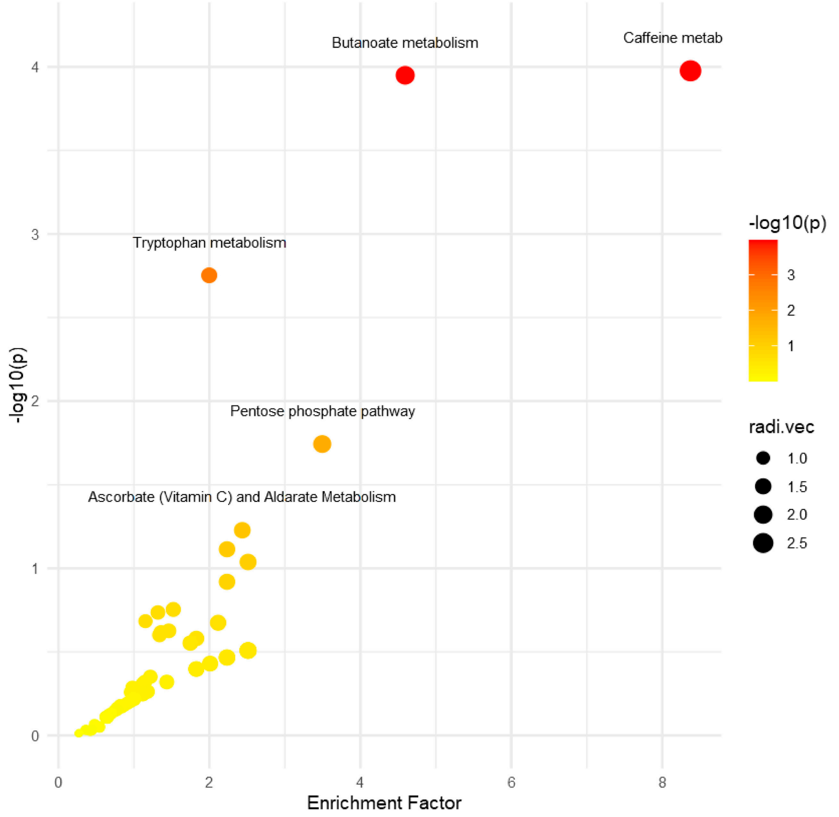

Supplemental Figure S3.

Baseline IL-6 quartiles

- Mean IL-6 before randomization
- Quartiles (2 and 3 combined to separate extremes):
  - Group 1: < 3.29
  - Group 2 : >= 3.29 and <4.99
  - Group 3: >=4.99

We found no association between baseline IL-6 quartiles and metabolomics profile.

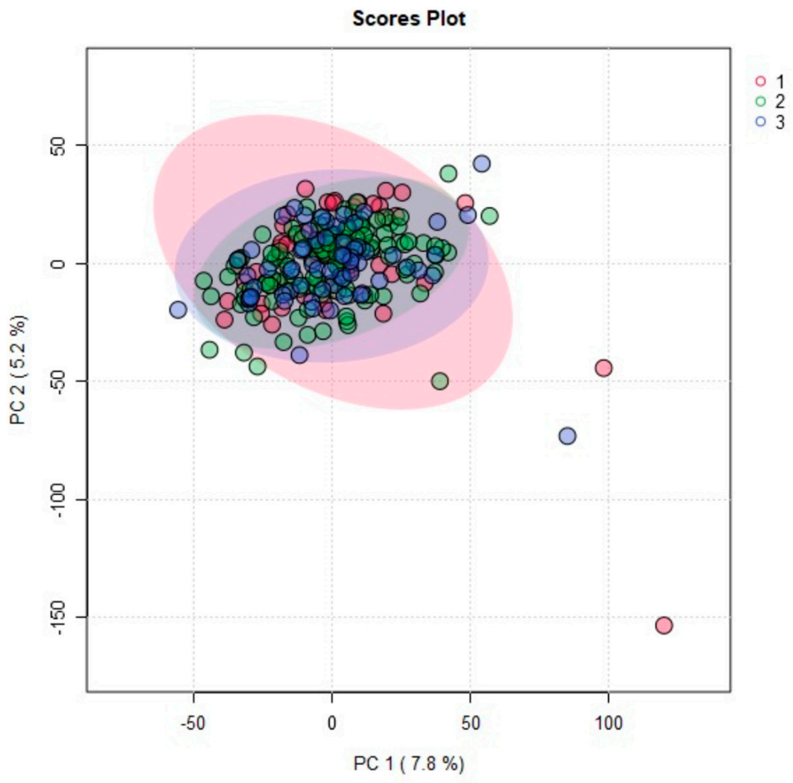

Supplement: Supplementary file 1 [file metabolites-16-00009-s001.zip › Supplemental Figures Metabolomics Manuscript+AEN (1).pdf]
